# A High-Efficiency Consultation Improves Urological Diagnosis in Patients with Complex LUTS—A Pilot Study

**DOI:** 10.3390/diagnostics13050986

**Published:** 2023-03-04

**Authors:** Alexandru Ciudin, Bernat Padulles, Pasqualino Manasia, Josep Alcoberro, Sanae Ounia, Maria Lopez, Natalia Allue, Joan Maria Ferrer, Jaume Duran, Antonio Aguilar

**Affiliations:** 1 Urology Department, Hospital Universitari de Mollet, 08100 Barcelona, Spain; 2 Hospital Management, Hospital Universitari de Mollet, 08100 Barcelona, Spain

**Keywords:** LUTS, diagnostics, uroflowmetry, ultrasound, cystoscopy, pressure-flow study, waiting list, time to diagnosis

## Abstract

(1) Background: The diagnosis of moderate-severe lower urinary tract symptoms (LUTS) is not easy due to the complexity of the micturition act. Sequential diagnostic tests can be time consuming due to waiting lists. Thus, we developed a diagnostic model combining all the tests in a single one-stop consultation. (2) Methods: In a prospective pilot study in patients with complex LUTS, they received all diagnostic tests (ultrasound, uroflowmetry, cystoscopy, pressure-flow study) in a single consultation and by the same doctor. Patients’ results were compared with those from a 2021 paired cohort that underwent the classical sequential diagnostic pathway. (3) Results: Per patient, the high-efficiency consultation saved: 175 days of waiting, 60 min doctor time and 120 nursing assistant time and over 300 euros on average. The intervention also saved 120 patient journeys to the hospital, lowering the total carbon footprint by 145.86 kg CO_2_. In one-third of the patients, performing all the tests within the same consultation contributed to reaching a more appropriate diagnosis and thus more effective treatment. Patients’ satisfaction was high, with good tolerability. (4) Conclusions: The high-efficiency urology consultation improves waiting times, therapeutic decisions and the degree of patient satisfaction while optimizing the use of resources and generating savings for the health system.

## 1. Introduction

Lower urinary tract symptoms (LUTS) are one of the main reasons for urological consultation. The prevalence of LUTS in patients attending a urology clinic was 41%, increasing with age: 14.1%, 41.5% and 60.8% of patients aged 18–49, 50–64 and ≥65 years, respectively. Of the 1015 selected patients, only 2.6% exclusively presented filling symptoms [1]. Lower urinary tract symptoms (LUTS) associated with benign prostatic hyperplasia are present in 20–30% of the Spanish male population aged 50 or over [2]. The prevalence of moderate/severe LUTS is 16.6% in men over 40 years of age (95% CI: 14.8–18.3) [2].

LUTS are common in women and can create a great deal of anguish and embarrassment, as well as considerable financial expenses for both individuals and society [3,4,5]. Prevalence estimates vary depending on the definition and the population under study. However, it is commonly agreed that the issue is crucial given the human suffering and financial costs involved. LUTS greatly modify a person’s quality of life, affecting their daily activities, modifying the things that one can do, limiting their ability to perform things, worsening night rest, modifying their mood and relationships with other people and obviously their ability to work and integrate into the world of work [6,7,8].

Not only can LUTS comprise filling symptoms (overactive bladder syndromes), voiding symptoms (bladder outlet obstructions such as BPH, urethral strictures or bladder-sphincter dyssynergia), urge incontinence, stress incontinence or even pelvic pain [6,9], but LUTS are also usually associated with a marked decrease in the quality of life, affecting the patient’s state of mind, which may affect their working productivity and integration or even impair their performance of daily activities [9]. Patients with complex moderate-severe LUTS tend to find it has a more marked impact on their quality of life than patients with mild LUTS. Therefore, the sooner a correct diagnosis is made and the optimal treatment is offered, the less time the patient will spend with a diminished quality of life [10].

The diagnosis is not easy, however, due to the complexity of the micturition act. The filling phase depends on bladder accommodation and detrusor relaxation and can cause irritative symptoms, usually classified as overactive bladder syndromes with increased urination frequency, urgency and even urgency urinary incontinence, sometimes associated with pain caused by the filling of the bladder. The emptying phase depends on bladder contractility (modified in hypocontractile detrusor), anatomical bladder outlet obstruction (BPH, urethral stenosis) or functional outlet obstruction (bladder–sphincter dyssynergia caused by suboptimal coordination of bladder contractility and sphincter relaxation) [11,12].

Given the simultaneous involvement of several organs and systems (bladder, prostate, pelvic floor muscles, nervous system) in complex voiding syndromes, several diagnostic tests are often needed to determine the reason for the appearance of symptoms [6,9].

The most common tests recommended in the EAU guidelines are an ultrasound, uroflowmetry, cystoscopy and urodynamic pressure-flow study [9]. Currently, the diagnosis process implies the performance of these tests separately in time and space, each one with its corresponding waiting time; the total waiting time for patients to have a complete final diagnosis can exceed 6 months.

To address the high prevalence of LUTS and the lengthy waiting time until a final complete diagnosis is available, we strongly believe that a high-efficiency urological consultation could offer an excellent solution.

On these bases, our hypothesis is that performing the recommended diagnostic tests in the course of one day, as part of one unique process, will have a positive impact on the quality of life, quality of diagnosis and treatment, and the health system. With those goals, the aim of this project was to develop a specific consultation for patients with complex LUTS who require various diagnostic tests. The objective of this consultation was to combine all the diagnostic tests in a single one-stop consultation, with the corresponding waiting time reduced. Our secondary objective was to demonstrate that performing all the tests at the same time provides a better approach, improving the diagnosis and treatment.

## 2. Materials and Methods

A prospective pilot study was conducted at our center between October and December 2022, implementing a high-efficiency consultation, with all diagnostic tests performed in a single consultation and by the same person for patients with complex LUTS.

Inclusion criteria (a + b/c/d + e): (a) age >18 years; (b) patients with two or more of the following symptoms in a moderate or severe manner: voiding symptoms, filling symptoms, urge urinary incontinence, stress urinary incontinence, pelvic pain; (c) operated patients with permanence of symptoms after surgery; (d) patients who did not respond to previous medical treatment; (e) signed informed consent form. Exclusion criteria: patients who refused to participate in the study.

To assess the benefits that this type of consultation could provide, a second cohort was created with the same number of patients and with similar characteristics, who visited the center in 2021 and following the standard of care clinical diagnostic protocol. The patients were selected based on the CIM 10 diagnostic codes, then they were matched by sex, age and initial symptoms with the patients evaluated using the high-efficiency consultation. In this cohort, we evaluated the waiting time of the patients (from the moment the need to carry out diagnostics was indicated in the urology office to the final complete diagnosis).

### 2.1. The Standard Clinical Protocol

The EAU guidelines recommend identifying differential diagnoses during urological diagnosis, since the origin of LUTS is multifactorial, and defining the clinical profile (including the risk of disease progression) of patients with LUTS in order to provide adequate care. To do so, the guidelines recommend using flowmetry, ultrasound, cystoscopy and/or urodynamics as complementary tests, without specifying a preestablished order or the need to use all or only some of the tests. So, based just on the evaluation in the urology office, the decision is made to expand the diagnostics by requesting one or more of the tests [6,9].

### 2.2. Ultrasound

The BK Ultrasound Flex Focus 400 ultrasound machine was used with a 5 Mhz abdominal probe. A standard kidney-bladder-prostate ultrasound was performed, evaluating the size and morphological and anatomical characteristics of the kidneys, urinary tract, bladder and prostate, along with the pre- and postvoid bladder volume.

### 2.3. Flowmetry

The MMS Solar System urodynamics machine was used. Free uroflowmetry was performed in a standing or sitting position according to the patient’s preference. We evaluated the maximum flow rate, voiding volume, voiding time and postvoid residue, as were assessed by ultrasound.

### 2.4. Cystoscopy

The CYF-VH cystoscope with the CV-170 image processing unit was used to perform a flexible cystoscopy according to the standard technique using water-based lubricant and continuous saline irrigation. The anatomy of the penile, membranous, bulbar, prostatic urethra, bladder neck and bladder was evaluated, providing information on the anatomical modifications, the length of the prostatic urethra, the degree of obstruction of the prostatic urethra, the bi- or trilobular growth of the prostate, the appearance of the bladder mucosa, the trabeculation of the bladder wall and the appearance of the trigone and the anatomical position of the meatus. Any pathological image was reported and diagnosed according to current protocols [13].

### 2.5. Pressure-Flow Study

The MMS Solar System urodynamics machine was used. A pressure-flow study was carried out in a standing or sitting position according to the patient’s tolerability, performing two fillings. Water pressure lines were used, performing atmospheric zero.

The following variables were also noted: the demographic data of the patients, the indication for a diagnostic study, the final diagnosis and the proposed treatment, the total time of the consultation, the average time until the final therapeutic decision, the influence on the therapeutic decision made at the end of the consultation, the savings for the hospital and health system, systemic savings obtained by reducing the number of visits and the degree of patient satisfaction.

The total time of the consultation was evaluated in order to define whether it is feasible to conduct all the indicated tests in one hour, which would be the time assigned to carry out the consultation and prepare the next one. This was compared with the average time assigned to each test and query separately, to assess whether there is a difference in favor of the high-efficiency consultation.

The average time until the final therapeutic decision was calculated from the visit when the need to expand the diagnostic study was indicated, either by performing a high-resolution consultation or following the classic sequential diagnostic algorithm.

The savings for the hospital and the health system were calculated by evaluating the costs of the extra tests or consultations that were saved by condensing them all into a single consultation and comparing this cost with the cost of the high-efficiency consultation. The cost of the material for the high-resolution consultation was considered to be similar to the other consultations since the material is the same, with differences only arising from the necessary personnel time.

We evaluated and compared the number of visits made by patients who underwent the classical successive diagnostic algorithm and patients who attended the one-stop high-efficiency visit. Furthermore, we evaluated if these patients came accompanied and the reduction in the number of visits that the high-efficiency consultation can produce for the family members that usually accompany patients.

The influence on the therapeutic decision was evaluated by comparing the therapeutic decision made after the high-resolution consultation with the decision that could have been made by performing the same tests sequentially. For this, the cases were presented to other urologists from the urology department who neither carried out the high-efficiency consultation nor indicated the diagnosis tests, in order to avoid bias. The tests were presented anonymously, in sequential order according to the diagnostic algorithm applied by the urologist who was evaluating the tests. It was not considered essential that the urologist requested all the tests be performed during the high-efficiency consultation, and the order of the tests was merely requested by the evaluating urologist. The diagnostic and therapeutic decision made by the evaluating urologists and the therapeutic decision made after the high-efficiency consultation were then compared.

The degree of satisfaction of the patients with having all the tests performed in the same consultation and the degree of pain caused by the tests were evaluated using Likert scales [14].

## 3. Results

A total of 30 patients attended the high-efficiency urology consultation at our hospital. Of those, 83.3% of the patients were male and 16.7% female. The average age of the patients was 66.5 (+/−15) years. Patients’ demographics can be found in Table 1. A total of 93% of the patients came accompanied by a family member, 53% by a retired one and 40% by a family member that needed to ask for a leave of absence from work. Flowmetry, ultrasound, cystoscopy and urodynamics were performed for all patients. In 26 cases, the indication was complex LUTS, whereas the remaining 4 patients were referred for a high-resolution consultation due to persistence of voiding symptoms after surgery (TURP).

As for the diagnoses after consultation, there were 3 urethral strictures, 3 pelvic floor myofascial syndromes, 12 BPH, 6 patients with hypocontractile detrusor, 2 bladder tumors and 3 idiopathic detrusor hyperactivities. The indicated treatments were: 3 internal optical urethrotomy surgeries, 9 TURP, 4 medical treatments for bladder outlet obstruction, 5 patients maintained their current treatment, 2 TURB and 1 botulinum toxin injection.

The average time for the high-efficiency consultation was 51 min (+/−7 min) versus 150 min for the classic sequential diagnostic algorithm.

When evaluating the 2021 cohort who were diagnosed according to the standard protocol, the four tests were performed in four different diagnostic consultations and one medical consultation. The personnel times required for each consultation are reflected in Table 2. When comparing the times of the sequential consultation with those of the high-efficiency consultation, it is shown that the high-efficiency consultation requires 60 fewer minutes for the doctor and 120 fewer minutes for the nursing assistant.

Since the materials used are the same, the differences between consultations are due to the staff time needed, creating an average saving of more than 300 euros per patient.

**Figure 1:**
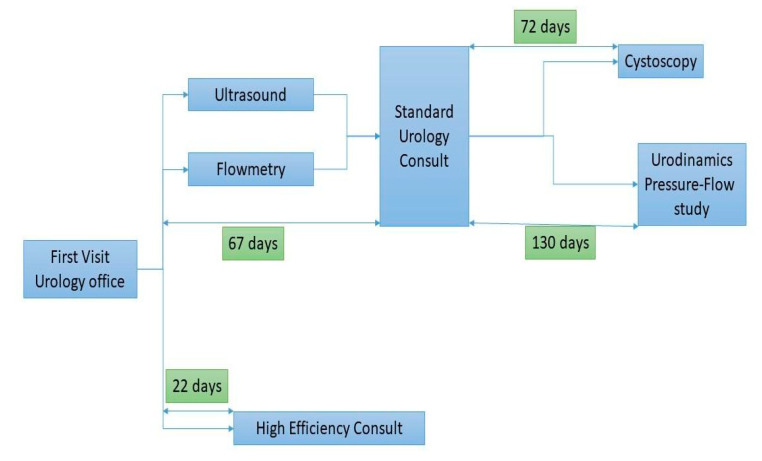
Comparative flowchart of the waiting times of the sequential diagnostic algorithm (above) and the high-efficiency consultation.

The average waiting time for the high-efficiency consultation of the patients was 22 days (±6 days), while the average time that the 2021 cohort needed to undergo all the diagnostic tests was 197 days (±31 days), with a mean difference of 175 days (Figure 1).

The number of visits to the hospital was reduced from five to one in favor of the high-efficiency consultation. From an overall point of view, the patients in the high-efficiency consultation made 30 trips to the hospital and the ones form the classical sequential diagnostic algorithm group made up to 150 trips, resulting in 120 less visits to the hospital. Furthermore, in 90% of the visits by patients in the sequential diagnostic algorithm group, they came accompanied by a family member, 46% by a retired one and 43% by a family member who required leave from work. In comparison, of the patients in the high-efficiency consultation, 93% came accompanied by a family member, 53% by a retired one and 40% by a family member who had to request a leave of absence from work. Family member visits were reduced from 135 to 28, and for family members who had to miss work, from 65 to 12 (Table 3).

In 33% of the patients, there were diagnostic and thus treatment differences between the urologists who evaluated the tests sequentially or in a high-efficiency consultation. There were six patients with obstructive syndrome due to a hypocontractile detrusor and an apparently obstructive image of the prostatic urethra, but with a non-obstructive urodynamic study; in these patients, TURP was proposed by the evaluating urologist but the fact that the pressure flow study ruled out bladder outlet obstruction changed the treatment decision. In addition, two patients were diagnosed with bladder–sphincter dyssynergia, and the urethral profile gained during the high-efficiency consultation changing the treatment from TURP to an initial medical and physiotherapeutic approach. Furthermore, there were two patients with irritative syndrome due to a bladder tumor, where cystoscopy changing the treatment from anticholinergic drugs indicated based on the pressure flow study to a TURB resection.

All patients reported being satisfied with the consultation (9/10 +/−0.6), with low pain levels caused by the four tests (3.7/10 +/−1.1). No complications were recorded after the consultation and no patient attended the emergency department or was diagnosed with UTI or hematuria.

## 4. Discussion

To our knowledge, ours is the first study to prove that optimizing the urological diagnostic process of patients with complex LUTS through a one-stop diagnostic consultation that includes all the necessary tests is an excellent way to reach an optimal diagnosis in the shortest possible time.

To date, there have been few attempts to optimize urological diagnosis through a one -stop consultation. The attempts were focused on patients received from primary care, not patients with complex LUTS [15,16,17]. We point out that not all patients referred to the urology office will need all the tests that can be performed in a high-efficiency consultation.

The diagnosis of moderate-severe LUTS is usually complex due to the mixture of voiding and filling symptoms, stress or urge incontinence and even pelvic pain. The complexity of the voiding act requires several tests for diagnosis, which may necessitate a long waiting time while the patient lives with a greatly diminished quality of life.

The few studies that tried to demonstrate the feasibility of a one-stop consultation did not focus exclusively on patients with complex LUTS, but on all patients sent from primary care to the urology office. Since moderate-severe LUTS represents only 16% of these patients, not all patients underwent all the diagnostic tests; in many cases, an ultrasound and uroflowmetry were sufficient. One of the questions raised in our study was whether it is feasible to carry out a high-resolution consultation focused only on patients with complex LUTS, knowing that all the tests must be performed in all patients. In our study, the average time for this consultation, including anamnesis, the four tests, explaining the test results to the patient, agreeing on the therapeutic decision and preparing the consultation for the next patient was 51 min, demonstrating that it is feasible to schedule the patients hourly.

The key goal of treating patients with LUTS is for them to regain quality of life and the ability to carry out activities of daily living, recover self-esteem and return to work. Therefore, the time between the start of the diagnosis study and the treatment is essential since patients live during this time with a low quality of life. In addition, patients with moderate-severe complex LUTS are the ones whose quality of life is most greatly impacted [18,19,20]. Our study shows that by carrying out a high-efficiency consultation, we can advance the diagnosis and the definitive therapeutic decision by up to 175 days—almost 6 months—in the case of complex patients.

Combining all the tests in a single consultation clearly saves time. A comparison between the consultation time for the 2021 cohort and the patients in the high-efficiency consultation showed that by implementing the high-efficiency consultation, up to 60 min of doctor time and up to 120 min of nursing assistant time can be saved. This can be translated either into savings of more than 300 euros per patient or into the possibility of specialists being able to see more patients due to time optimization.

Our study demonstrates that the high-efficiency consultation can reduce the number of trips to the hospital, not only for the patients but also for their family members. The number of visits needed for each patient was reduced from five to one. Therefore, 120 trips to the hospital were avoided. The mean age of our patients was 65 years, and most of them came accompanied by a family member. The reduction in trips to the hospital was also seen in case of family members from 135 to 28, and even more importantly, the number of family members that had to miss work was reduced from 65 to 12.

From an environmental point of view, reducing the number of visits and trips to the hospital lowers the carbon footprint that these patients generate. Our study avoided 120 visits to the hospital, and considering that the average distance our patients live from the hospital is 7.15 km, a total of 858 km were saved. A study carried out in a similar setting quantified the amount of CO_2_ saved by avoiding a kilometer of travel to the hospital as 0.17 kg CO_2_/km; therefore, in our case, the carbon footprint was lowered by a total of up to 145.86 kg CO_2_ [21,22].

There are many treatment alternatives for LUTS, and in some cases, they have opposite effects and results [6,9]. For this reason, a precise diagnosis must always be made before deciding on treatment, sometimes even balancing the resolution of one type of symptom with that of another, especially in patients with severe filling and voiding symptoms.

Our study shows that performing all the tests jointly can improve the integration of diagnostic data by the health professional and, therefore, optimize the diagnosis and the proposed treatment. In one-third of the patients, performing all the tests within the same consultation contributed to reaching a more appropriate diagnosis and, therefore, offering more effective treatment to the patient.

**Figure 2:**
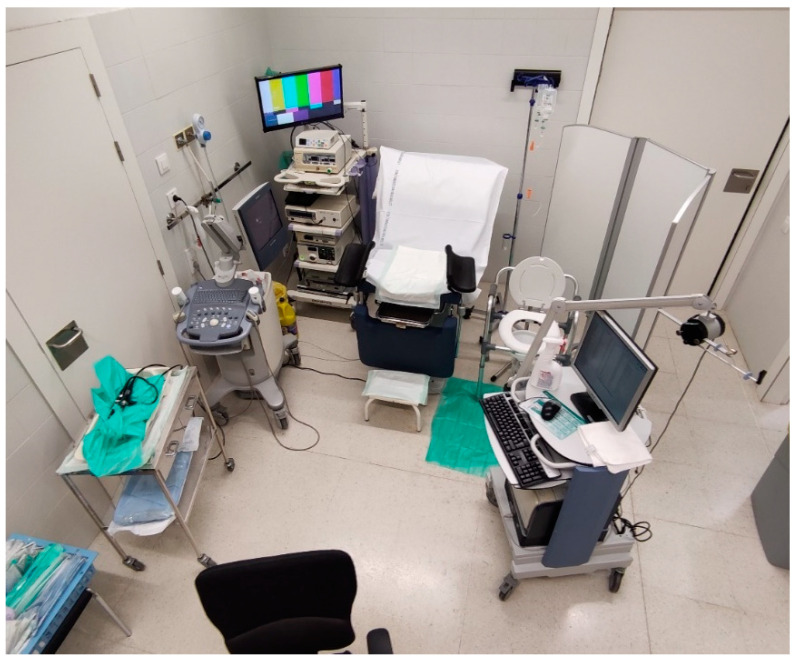
The high-efficiency consultation setting.

One of the initial obstacles when developing the concept of high-efficiency consultation was the space where it could be carried out. Due to the high number of tests that needed to be performed in a limited time, there were concerns about whether the consultation could be carried out in the space of a standard diagnostic and examination office. However, through an ergonomic arrangement of the elements involved (examination table, ultrasound, additional table for cystoscope, urodynamic machine), a space (Figure 2) was arranged where all tests could be performed effortlessly. A key element in achieving a smooth consultation was having expert, trained personnel with solid knowledge of all the issues involved, which made carrying out the consultation efficient.

One of the limitations of our study was the small number of patients. However, this study was designed from the beginning as a pilot test to support the subsequent performance of a larger study or to modify the usual clinical practice. The relatively small number of patients does not prevent us from underlining the advantages that a high-efficiency consultation could provide both for patients and the healthcare system.

Last but not least, there was concern as to whether carrying out all the tests in the same consultation would be poorly tolerated by the patients. The results of the questions on tolerability in our study showed that the consultation was well tolerated by the patients, without complications and with high degrees of satisfaction.

## 5. Conclusions

A high-efficiency urology consultation is a feasible alternative to traditional successive consultations, improving waiting times, the therapeutic decision and the degree of patient satisfaction while optimizing the use of resources and generating savings for the health system. The results obtained since its implementation have been excellent, paving the way for an evolution toward a more efficient health system in all aspects, as well as patient-centered care that allows greater satisfaction.

## Figures and Tables

**Table 1:** Patients’ demographics.

	Study Cohort	2021 Cohort
Number of patients	30	30
Male %	83.3%	83.3%
Average age	66.5 years	67.2 years
Number of diagnostic visits per patient	1	5
Time required for diagnosis	51 min	150 min
Mean waiting time	22 ± 6 days	197 ± 31 days

**Table 2:** Staff time required for sequential query versus high-efficiency consultation.

	Sequential Diagnostic Algorithm		High-Efficiency Consultation	
	Medical Time	Nursing Assistant Time	Medical Time	Nursing Assistant Time
Ultrasound	20 min	20 min	60 min	60 min
Flowmetry	-	30 min		
Cystoscopy	30 min	30 + 30 min		
Urodynamics	60 min	60 min		
Medical consultation	10 min	10 min		
Total	120 min	180 min	60 min	60 min

**Table 3:** Reduction in number of visits to the hospital.

	Study Cohort	2021 Cohort
Total number of visits to the hospital (all patients)	30	150
Patients who came accompanied	93%	90%
Number of visits for accompanying persons	28	135
Number of visits for working accompanying persons	12	65
Total km	214.5 km	1072.5 km
CO_2_ footprint	36.46 kg CO_2_	182.32 kg CO_2_

## Data Availability

Data is not available due to the Spanish Organic Law on Data Protection.
